# Morphological Characterization of Emerging Cercariae among Lymnaeid Snails from Barangay Cawongan, Padre Garcia, Batangas, Philippines

**DOI:** 10.1155/2018/5241217

**Published:** 2018-09-10

**Authors:** Gregorio L. Martin I, Esperanza C. Cabrera

**Affiliations:** ^1^University of Santo Tomas, Graduate School, Manila, Philippines; ^2^Department of Biology, De La Salle University, Manila, Philippines

## Abstract

**Background and Objectives:**

Lymnaeid snails are the known intermediate hosts of the liver fluke* Fasciola* spp. and therefore play an important role in the parasite's life cycle. The study is conducted to determine specificity of snail host-parasite interaction and to determine the snail-trematode infection rate by cercarial emergence, characterizing the emerging larvae using standardized key.

**Materials and Methods:**

A total of 750 snails were collected from a rice field in Barangay Cawongan, Padre Garcia, Batangas, from November 2016 to March 2017 (n=150/month). Preliminary snail identification was based on morphological features of the shell. Each snail was acclimatized for 24 hours in a 50-ml capacity container before being exposed to strong artificial light. The 150 snails collected per month were grouped into 5 batches (n=30/batch) with each batch receiving different number of light exposures. Emerging cercariae were described and characterized using photo-referencing and standardized keys. All statistical tests were performed at p<0.05 level of significance using SPSS ver. 20.

**Results:**

The total cercarial shedding rate of the snails studied, as a measure of the infected snails, was found to be 35.6% and was positively associated with the length of the snail shell [OR = 1.809; 95% CI: 1.471–2.225; p<0.001], but not with the weight [OR = 0.003; 95% CI: 0.00–0.275; p=0.012] and width of the shell [OR = 0.937; 95% CI: 0.672–1.305]. The rates varied from 29.3% to 38.0% based on the frequency of 6-hour light exposure. Appearance of encysted forms increased with increasing number of light exposures [OR = 10.27, 95% CI: 3.04–34.76, p<0.001]. Three distinct cercariae were identified, namely, echinostome, longifurcate-pharyngeate distome cercariae (*Strigea* cercariae), and the virgulate xiphidiocercaria, with 26.4%, 2.27%, and 0.67% infection monitored by cercarial emergence, respectively.

**Conclusion:**

Local lymnaeid snails were infected with a single type of trematode larvae and coinfection with multiple larvae was rare but was encountered.

## 1. Introduction

Freshwater gastropods exist as important components of aquatic food webs. They also serve to balance the ecological niche by providing nutrients to both land and water ecosystems [1]. Their population increases, and their fecundity is affected by natural changes as seen in climate change [2], physical change as observed in creation of cemented damps, and chemical exposures through chemical sprays used in crop farming [3, 4]. In a different context, they are considered miniature nuisances that affect some agricultural products [4] and serve as intermediate hosts to human and animal parasites [3, 5].

Molluscs under the family Lymnaeidae are given much attention as they participate in the life cycles of many trematodes with large-scale biomedical and veterinary significance [5, 6]. In fact, they are distributed worldwide and are known to act as intermediate hosts of more than 71 species of flukes belonging to 13 different families [7]. Larvae that are known to infect lymnaeid snails belong to superfamilies Schistosomatoidea, Fasciolidae, Clinostomoidea, Paramphistomoidea, Echinostomatoidea, Diplostomoidea, and Pronocephaloidea [8], which cause diseases when transmitted to humans and animals [9].

Snails belong to the large group of class Gastropoda which has been recently reclassified by Bouchet and Rocroi (2017) into eight (8) distinct subclasses namely: subclass Amphigastropoda, subclass Archaeobranchia, subclass Patellogastropoda, subclass Neomphaliones, subclass Vetigastropoda, subclass Neritimorpha, subclass Caenogastropoda, and subclass Heterobranchia [10].

There are 11 different families of freshwater gastropods with 26 genera reported in the Philippines [11]. Among the families are Neritidae (Rafinesque 1815), Viviparidae (Gray 1847), Ampullariidae (Guilding 1828), Bithyniidae (Gray 1857), Pomatiopsidae (Stimpson 1865), Stenothyridae (Fischer 1887), Paludomidae (Gill 1871), Thiaridae (Troschel 1857), Lymnaeidae (Rafinesque 1815), Planorbidae (Rafinesque 1815), and Ancylidae (Rafinesque 1815). These snails belong to phylum Mollusca, which is considered to be an extremely diverse taxon [12], with about 100,000 species described worldwide [13]. However, roughly less than 5% of the world's gastropod fauna are freshwater snails, which inhabit diverse forms of aquatic reservoir like rivers, streams, ponds, canals, and swampy rice fields [13].

Despite their abundance, freshwater molluscs are rarely studied. To date, local studies have been limited to their focal biogeographical distribution, biodiversity, and inventory [14–17] and to snail identification using varied conchological parameters and molecular attempts [18, 19]. Very few data on snail host parasite interaction have been recovered, and these were mainly focused on* Oncomelania hupensis quadrasi, *due to the utmost interest in the nationwide control of schistosomiasis caused by* Schistosoma japonicum*. Studies on infection rates of* O. hupensis quadrasi *have been used as adjunct data for epidemiologic profiling of the extent of the blood fluke infection in man [20]. In this context is the study being conducted to explore parasite diversity in other freshwater gastropods like the lymnaeid snails, which are known to harbor* Fasciola *sp. parasite, a medically important parasite that causes liver fluke infection among humans and cattle. Pulmonates like planorbids and lymnaeids are among the most important intermediate hosts involved in a fluke life cycle where vertebrate hosts like mammals and birds are commonly parasitized [21]. However, the Philippines lacks records on the varieties of parasites inhabiting different snail intermediate hosts. Currently, there are no recent or comprehensive studies on diversity of trematode larvae inhabiting our local lymnaeid snails.

Snail-parasite interaction is a dynamic and highly specific phenomenon that is required for the development of the larval stages of certain parasites, usually those belonging to the class Trematoda. Trematodes, whether monogenetic or digenetic, will always require snails in their complex life cycles. The miracidium, upon release from the egg, will infect a susceptible snail host and internally will continue to develop into sporocyst form, transforming into the redia where a mass of cercarial stages is enclosed. The motile cercariae will then be released from the snail and will initiate encystment on a suitable second intermediate host as infective metacercariae [22]. These various intramolluscan stages undergo polyembryony and affect the growth pattern of the infected host, influencing its physiology, metabolism, fecundity, and survival rate [23, 24]. These five larval stages are commonly observed among nonschistosomal parasites. Infection is considered to be territorial as no other parasite species should and can infect a previously infected snail host [25]. This concept of territory may therefore provide an avenue for control of the transmission pattern of potentially harmful parasites like that of the* Schistosoma *sp. and* Fasciola *sp. Nonhuman pathogen infecting the same intermediate host may provide safe models for biodiversity and biocontrol [1]. Literature search showed that no study has yet been done in the Philippines on parasite symbiosis or coinfection. Hence, this study aims to determine parasite symbiosis or coinfection in the snail host and to determine trematode infection rate in the snails by cercarial emergence through intense artificial light stimulation. Moreover, emerging larvae were characterized to provide initial parasite grouping and identification in order to provide impetus to future epidemiological survey of snails with medical and veterinary importance.

## 2. Materials and Methods

### 2.1. Snail Sampling and Preliminary Identification

**Figure 1:**
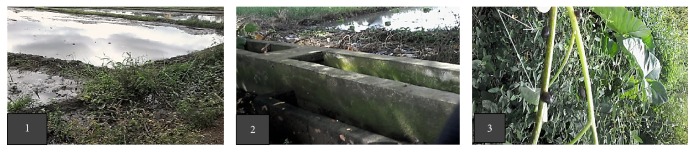
Sampling site. (1) Overview of the rice field flooded with water scheduled for a new batch of plantation. (2) Portion of the water drainage system of the area. (3) Lymnaeid snails attached to a portion of the stem of* Ipomea aquatica*.

A total of 750 lymnaeid snails (n=150/month for 5 months based on the computed sample size [26]) were collected from the waters of a rice field and areas near the water drainage in Barangay Cawongan, Padre Garcia, Province of Batangas, Philippines (Figures 1(1) and 1(2)). Padre Garcia is located at 13.88° North latitude, 121.21° East longitude, and 182 meters elevation above sea level. This fertile area also abounds with* Ipomea aquatica *(or* kangkong*) which provides a favorable habitat for the growth and survival of the snails. Some snails were collected from the stem of the* kangkong *plants where they were found attached (Figure 1(3)). Snails were handpicked individually and placed in a big plastic containment. They were then placed in a plastic strainer and were dipped several times in a basin of spring water to remove macro-debris and muddy substance that adhered onto the snail surface. Collection was conducted from 8:00 to 10:00 AM once in a month from November 2016 to March 2017. Preliminary snail identification based on morphological features of the shell was done using standardized taxonomic keys as described by Burch (1980) [11] and by photo-referencing of images from varied journal articles.

### 2.2. Snail Segregation and Cercarial Emergence

After washing the snails on site, they were immediately placed individually in 50-ml capacity containers with perforated covers to prevent the captive snails from escaping and to provide them with good aeration [1]. Each container had approximately 10-15 ml natural spring water with a neutral pH. They were acclimatized for 24-hours before exposing them to artificial light with an average of 992.2 ± 288.5 lux as determined using light meter HS1010. The 150 snails collected per month were grouped into 5 batches (n=30/batch) with each batch receiving 1, 2, 3, 4, and 5 times of light exposure in an improvised illuminated cabinet (refer to Supplementary Materials available here). Light exposure started at 8:00 AM and ended at 2:00 PM every day for the assigned number of exposures. Temperature and relative humidity were also recorded. Each exposure was for 6 hours to ensure the emergence of the cercariae of most species of flukes of medical and veterinary significance [27, 28]. Repeated exposure with the same light intensity identified possible variation in cercarial emergence and metacercarial (encysted larvae) formation over time, and the significant difference was statistically calculated.

### 2.3. Microscopic Examination of Emerging Cercariae and Observation of the Presence of Encysted Larvae

Liquid sample from each snail set-up was centrifuged at 2500 x g for 5 minutes using Clay Adams™Dynac III. The supernatant was collected for pH determination (Milwaukee pH600 tester), and the sediment was examined using Olympus CX21. Motility and morphology of live, unstained cercariae that emerged from the snails were noted to identify these to the major type level [29]. Initial identification of emerging cercariae was done using standardized taxonomic keys [27]. Representative of each type of emerging cercaria was subjected to scanning electron microscopy (TM3000 Hitachi Tabletop Microscope). Presence of round cyst-like bodies was also noted across different light exposures and was statistically correlated.

### 2.4. Snail Dissection and Morphometrics

The flesh of each snail was carefully removed from the shell, and the foot tissue was dissected using a sterile surgical blade. The foot and soft body tissue were placed separately in microfuge tubes and preserved in 70% ethanol for further studies. Shell morphometrics were determined by measuring the length and width using Tactix 245111 digital Vernier caliper. Weight of empty shell was determined using Shimadzu TX223L analytical balance. Snails were wiped first with filter paper and dry cotton prior to weighing to remove excess liquid and to remove adhering debris. Significant correlation of snail infection rate and the snail morphometrics was statistically calculated.

### 2.5. Statistical Analysis

The infection rate of the snails was determined by the percent of snails with emerging cercariae. Multivariate logistic regression was performed to determine if the months of collection, number of light exposures, and snail morphometrics were predictors of infection rate. All tests were performed at p<0.05 level of significance using SPSS ver. 20.

## 3. Results

### 3.1. General Appearance of Lymnaeid Snail

**Figure 2:**
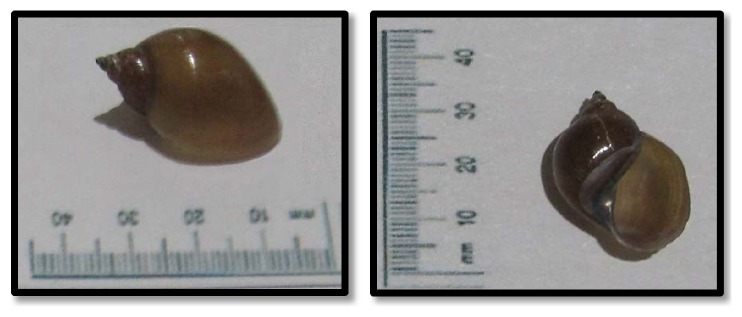
Lymnaeid snail identified in the study.

The collected snails resembled lymnaeid snails (Figure 2). They had light to dark brown shells with four whorls and a pointed prominent tip. The shell surface demonstrated transverse growth lines or striae. Apertural opening was dextral and body whorl was enlarged. Body whorl was expanded and had a twisted columellar lining. These measured an average length of 12.94 mm ± 1.72 mm and an average width of 7.26 mm ± 1.01 mm. Empty shell weighed 0.05 g ± 0.1 g. Shell generally appeared oval broadly and nearly globosely conical in shape. Despite minor differences, the snails in the current study were identified as* Lymnaea *(*Radix*)* quadrasi*.

### 3.2. Emerging larval Forms

Cercariae are the terminal intramolluscan larval stages that emerge from an infected snail host. They may be readily infective to some mammalian hosts or may encyst in a susceptible second intermediate host. Recovery from the samples is usually done through strong artificial light exposure that triggers their release, and the classical method to identify them is through morphological characterization using standardized taxonomic keys [30]. Our current set-up made use of an artificially illuminated cabinet (refer to Supplementary Materials) with a light source that emitted approximately 1000-lux intensity. This was enough to allow release of cercariae from the snails. Results of the study showed that the actual rate of cercarial shedding can be established using a single 6-hour light exposure at this intensity, as this did not differ among snails exposed for more than once up to five times.

Three cercarial forms were observed to emerge from the snails (Figures 4 and 5).  Table 1 shows the frequency of recovery of these different types from the lymnaeid snails studied.

#### 3.2.1. Echinostome Cercariae

**Figure 3:**
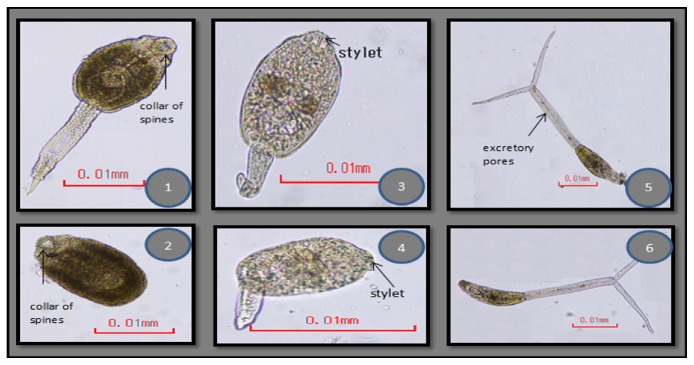
Microscopic images of different types of cercariae that emerged from lymnaeid snails (CX 21, 400x magnification). (1)-(2) Echinostome cercariae. A collar of spines in the oral sucker is noted. Upper image shows the complete larvae, while the lower image shows the de-tailed part of the body. (3)-(4) Virgulate xiphidiocercaria. Take note of defined stylet present in the anterior-most part of the oral sucker. (5)-(6) Longifurcate-pharyngeate distome cercariae (*Strigea* cercariae). Excretory pores are seen along the midportion of the bifurcated tail.

**Figure 4:**
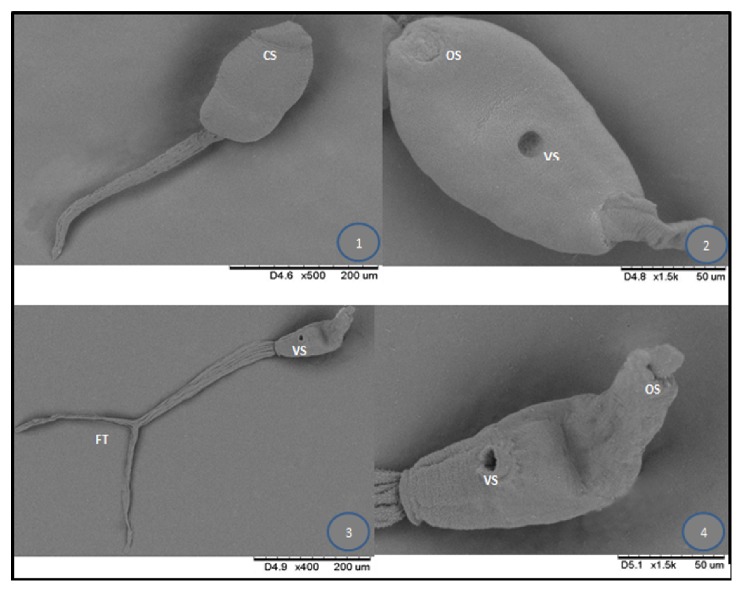
SEM images of three distinct cercariae that emerged from lymnaeid snails. (1) Echinostome cercaria showing a crown of spine (collar of spines). (2) Virgulate xiphidiocercaria. (3)-(4) Longifurcate-pharyngeate distome cercariae (*Strigea* cercariae). OS: oral sucker, VS: ventral sucker, FT: fork-tailed, CS: collar of spines.

**Figure 5:**
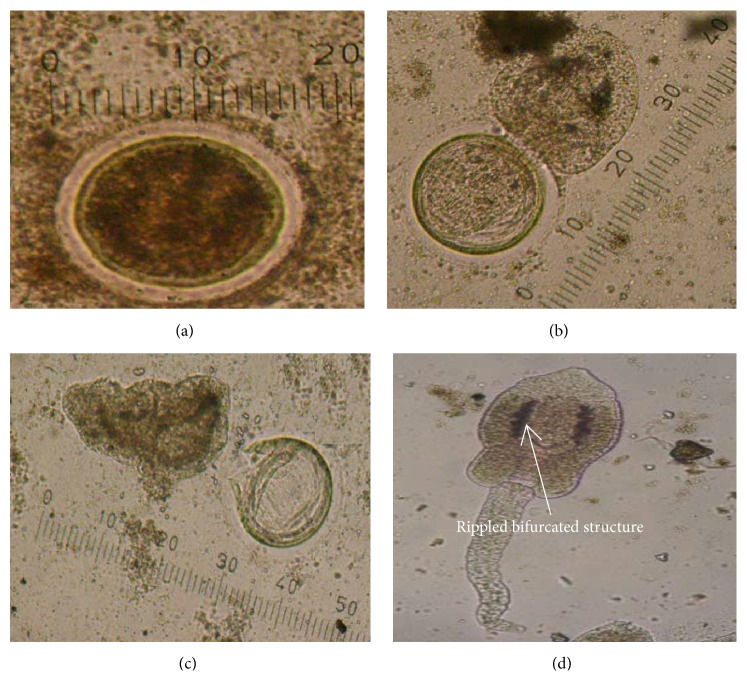
Development of an echinostome larva released from an unknown encysted form. (a) Encysted larval form. (b) Partial emergence of larva showing its head. (c) Larva newly emerged from its encysted form. (d) Fully emerging larva complete with its head and tail. Rippled bifurcated structure shown.

**Figure 6:**
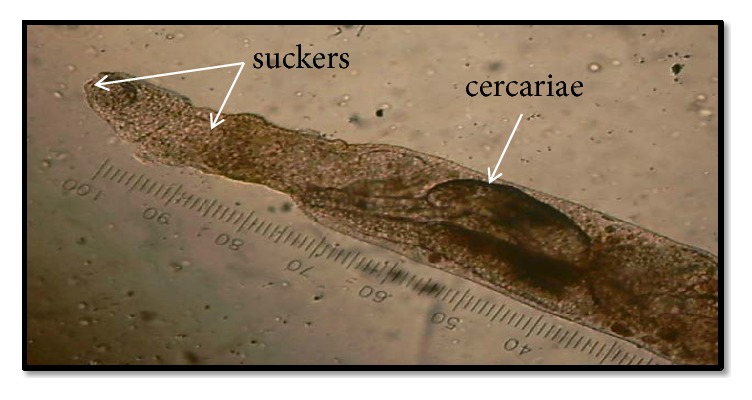
Redia stage with echinostome cercariae inside (upper half of the whole redial body shown) (CX21400x magnification).

The echinostome type of cercariae was found in 26.4% of the 750 snails studied and was the most common among the three types of cercariae (Figure 3: (1)-(2)). Freshly emerging larvae moved in a twitching manner using their powerful tails which eventually detached themselves from the body. The elongated oval-shaped body then moved in a slow crawling motion. The whole larvae measured an average size of 30 *μ*m ± 0.6 *μ*m x 11.4 *μ*m ± 2.1 *μ*m. The slender unforked tail was as long as the body and measured on the average 31 *μ*m ± 9.0 *μ*m x 4.5 *μ*m ± 0.7 *μ*m. Two prominent suckers were found to be present. The oral sucker was in the anterior subterminal part of the body and was crowned with spinous processes as revealed from the scanning electron microscope (Figure 4). The number of spines in the circular area was difficult to ascertain. The ventral sucker was slightly below the midportion of the body. Dark rippled bifurcated structures (Figure 5(d)) were observed from within the body, which were continuous from the oropharynx and may represent esophageal tubing. Figures 5(a)to 5(d) show echinostome larva that was found to be emerging from an unknown encysted form. This observation has not been reported in published literature before.  Figure 6 is a mature redia with multiple live cercarial forms.

#### 3.2.2. Virgulate Xiphidiocercaria

Virgulate xiphidiocercariae is another type of cercaria that was recovered from the lymnaeid snails (Figure 3: (3)-(4)). However, this was only evident with the February 2017 and succeeding batches of samples. The tail had no dorsoventral finfold, but the cercariae were observed to move in a gentle sweeping motion. Bilobed or pyriform virgula organ was present in the oral sucker, and a distinct stylet was observed. Ventral sucker was smaller than the oral sucker. Micrometry of representative samples revealed that this kind of cercaria measured an average body size of 28 *μ*m x 12 *μ*m and an average tail size of 25 *μ*m x 4 *μ*m. Parasites with this type of cercaria belong to the family Lecithodendriidae and are known to infect intestines of bats, birds, and amphibians. Out of the 750 samples collected for the whole 5 months, 0.67% were infected with this kind of cercaria.

#### 3.2.3. Longifurcate-Pharyngeate Distome Cercariae (*Strigea* Cercariae)

The presence of fork-tailed cercariae (Figure 3: (5)-(6)) has not been reported yet among snails in the Philippines other than* Oncomelania hupensis quadrasi*, a known snail intermediate host for* Schistosoma japonicum*. The emerging cercaria was twice as long as the typical cercaria of* Schistosoma japonicum*. Its head measured an average of 41 ± 1.4 *μ*m x 9 ± 1.4 *μ*m and the tail body measured an average of 34.7 *μ*m ± 4 *μ*m x 5 *μ*m. Half of its tail measured on the average 20.3 *μ*m ± 0.6 *μ*m x 2 *μ*m. Two suckers were observed and these were subequal in size. It may be confused with a brevifurcate-distome apharyngeate cercaria, but the presence of distinct excretory pores within the tail stem proved otherwise. Out of the 750 snails collected, 2.27% were infected with this type of larva. This type of cercaria belongs to the families Strigeidae and Diplostomidae which parasitize birds and mammals.

### 3.3. Presence of Coinfection among Lymnaeid Snails

Existence of coinfection is a very rare phenomenon among lymnaeid snails. Snails coded 3FM35 and 1 M61 demonstrated the presence of both echinostome and fork-tailed cercaria. This represented 0.27% of the total 750 snails collected. 1F94-coded snail showed an echinostome and xiphidiocercaria, and this represented 0.13% of the total 750 snails collected. On the other hand, it is noted that 5.9% of the 750 snails showed only encysted forms.

### 3.4. Snail Infection in Different Months of Collection and Number of Light Exposures

The infection rate was determined by monitoring the number of snails that shed cercariae. Of the 750 samples collected within the 5-month sampling period, 267 or 35.6% [35.6%; 95% CI: 32.2 to 39.1%] shed cercariae. The distribution of infected samples based on the month of collection and number of light exposures is shown in Table 2. Generally, the rate of infected snails shedding cercariae differed according to the month of collection, with snails collected in February 2017 showing the highest infection rate at 57.3%, followed by those in January 2017 (*p*<0.001). The infection rates for snails collected in November 2016, December 2016, and March 2017 were significantly lower than those in the aforementioned two months, but did not differ significantly from each other (p>0.05). On the other hand, the rate of cercarial shedding among snails did not differ according to the number of light exposures (*p *= 0.513). In other words, actual rate can be established from a single 6-hour light exposure at an average of 992. 2 ± 288.5 lux (95% CI: 703.7–1280.7 lux). Nonshedding snails may mean any one of two things: (1) they were not infected with any trematode parasite; (2) they were infected but with immature precercarial larval forms which were not yet ready for release. However, these were not further determined using microscopic examination of crushed samples.

### 3.5. Association of Snail Morphometrics with the State of Infection

The shell length (mm), width (mm), and weight (g) were measured and were associated with the state of infection which was monitored and established by cercarial shedding.

Results in Table 3 show that 1 mm increase in length leads to an increase of 80.9% in the odds that the snails were infected [OR = 1.809; 95% CI: 1.471–2.225;* p*<0.001]. Longer snails tended to be infected as compared to shorter snails. On the contrary, one gram increase in shell weight led to a decrease of 99.7% in the odds that a snail is infected [OR = 0.003; 95% CI: 0.00–0.275;* p*=0.012]. Although the observed difference in the average weight of the shell was slight between infected and uninfected snails [average shell weight (infected) = 0.11 ± 0.01 > average shell weight (uninfected) = 0.09 ± 0.01], logistic regression of the raw data showed that, statistically, the heavier the snail's shell, the lower the chance that it was infected. Snail width on the other hand, [OR = 0.937; 95% CI: 0.672–1.305] was not a predictor of the state of infection, despite the difference in the average width of infected snails and the uninfected ones [average width (infected) = 7.59 ± 0.06 > average width (uninfected) = 7.07 ± 0.05]. Meanwhile, pH values of the 600 water samples in which the snails were placed (infected: mean ± SEM= 8.20 ± 0.01, n = 222; uninfected: 8.23 ± 0.01, n = 378) were not found to be predictors (*p*=0.150) of the state of infection. Therefore, presence of snail infection as measured by cercarial emergence may be predicted by the length, but not by the gross shell weight and width of the snails.

### 3.6. Development of Encysted Forms from Cercariae

The water in the set-ups where the snails were placed was examined for the presence of encysted forms after the different light exposures.  Table 4 shows the number of snails with encysted forms in the water, while Figure 5 shows the development of an echinostome larva released from an unknown encysted form.

Early light exposures on 24-hour acclimatized snails did not trigger encystment process, although some deaths were recorded. A total of 79 out of the 750 snails studied [10.5%; 95% CI: 8.4 to 13.0%] demonstrated encysted forms in the water after varying number of light exposures. These appeared as round bodies (Figure 6) that measured about 20 *μ*m in diameter. An irregular transparent covering extended 2 *μ*m from the outermost membrane.

There were significantly increasing odds (*p*<0.001) of producing encysted form as number of light exposures increased. Although one and two light exposures did not significantly increase the rate of encysted forms [OR = 2.76, 95% CI: 0.72–10.62;* p*=0.139), three [OR = 7.98, 95% CI: 2.33–27.36;* p *= 0.001], four [OR = 7.98, 95% CI: 2.33–27.36;* p *= 0.001], and five [OR = 10.27, 95% CI: 3.04–34.76,* p*<0.001] light exposures significantly increased the odds of having encysted form. It could be that encysted forms more commonly occurred after longer time of exposure of snail hosts as a response of the parasite to the environment that has become unfit for survival. This observation coincided with the increasing mortality observed over time.

## 4. Discussion

### 4.1. Identification of Lymnaeid Snails

The present study explored lymnaeid snails that thrived in the rice field of Barangay Cawongan, Padre Garcia, Batangas. They have been primarily implicated in the transmission of liver flukes in the Philippines [31], but have also been associated with local cases of human echinostomiasis [32]. They naturally support the life cycles of* Fasciola *spp. that cause diseases in majority of the ruminants like cows and carabaos [33]. The prevalence of human fascioliasis in the Philippines may be low, but potential transmission of other parasitic infections with medical importance like echinostomiasis [32] and avian schistosomiasis [34] cannot be discounted, if cercariae of these parasites are likewise found in the same lymnaeid snails. These non-*Fasciola *infections are caused by parasites whose other known intermediate hosts are nonlymnaeid snails, which in the Philippines include* Pila luzonica*, second intermediate host for* Echinostoma ilocanum*, and* Oncomelania hupensis quadrasi*, first intermediate host of* Schistosoma japonicum*.

There are three recognized genera under the family Lymnaeidae: genus* Austropeplea *(Cotton 1942), genus* Bullastra *(Bergh 1901), and genus* Radix *(Montfort 1810) [11].* Lymnaea *was not included among these. In many reports, all lymnaeid snails in the Western Pacific belong to a single Holarctic genus,* Lymnaea*. Confusion in snail identification and taxonomy has been known for a long time and is common among lymnaeids [35].

The snails in the study were tentatively identified as* Lymnaea *(*Radix*)* quadrasi *based on the morphological features of the shell using standardized taxonomic keys as described by Burch (1980) [11] and photo-referenced with images from varied journal articles. They bore close resemblance to those of* Lymnaea *(*Radix*)* rubiginosa *of Indonesia and Thailand based on their morphological characteristics [36]. Snail identification through gross description of the shell appearance and internal structure detailing is difficult and may be extremely confusing [37]. These taxonomic problems were already evident in various studies which dealt with several stagnicoline snails [7, 38, 39]. Confusions had been made on adult* Galba truncatula*, which was mistaken as preadult* Lymnaea palustris *or* Lymnaea fuscus*. The ever-changing environmental conditions have affected the general population of various living snails. Snails have their own adaptive mechanisms that allow morphological variations to happen among them. Consequently, this has made identification of snails more difficult [40]. In addition, shell shapes may vary as a result of natural trematode infection, and dilemma in phenotypic characterization consequently happens [41]. Despite this, there are still few attempts to study the phylogeny of lymnaeids in Asia. The same dilemma was encountered in the identification of the 750 collected snails. Based on shell features in standardized keys and photo validation, they were consistent with* Lymnaea *spp. However, other published references suggest that they belong to the Genus* Radix*. The snails demonstrated slight variations in shell coloration but were distinct in terms of apertural opening, number of whorls, and other significant morphometrics. Despite these inconsistencies in phenotypic identities, what is definite is that these snails belong to family Lymnaeidae, and molecular confirmation is currently being undertaken using* COX-1 *gene primers to resolve this inherent problem [42].

### 4.2. Types of Cercariae from the Lymnaeid Snails

Three different types of cercariae were observed to have emerged from the snails in the study. These were single infection of echinostome cercariae, which were predominant (26.4%), followed by longifurcate-pharyngeate distome cercariae (2.27%) and virgulate xiphidiocercariae (0.67%), which was only evident in snails from the latter part of the monthly collection. It is noted that no lymnaeid snail was found to harbor the gymnocephalus cercaria that is typical of* Fasciola *spp., the parasite for which* Lymnaea *spp. are known to be the common intermediate hosts. The absence of the* Fasciola *cercariae from the lymnaeid snails is strongly suggestive of the absence of inherent fascioliasis in the area. The number and types of definitive hosts that are present in the area can influence the nature of infections. The possibility of fascioliasis in the area is low because the number of definitive hosts not known to harbor the adult* Fasciola *spp. parasite is high. That is, the presence of other significant hosts like ducks, chickens, wild bird-dwellers, and amphibians which may harbor different parasites is evident from the area and may provide a rational explanation for the existence of these other types of cercariae. This supports the dilution effect hypothesis that underscores the relevance of negative correlation between disease incidence and host diversity [43]. Arguably, this hypothesis may not be always consistent in most human infections with great public health impact [44]. The finding of larvae other than* Fasciola *has likewise been reported by Faltynkova et al. [45], where they reported 26.3% prevalence among* Lymnaea stagnalis *of various kinds of larvae identified to be* Opisthioglyphe ranae*,* Plagiorchis elegans*, and* Echinoparyphium aconiatum. *On the other hand, furcocercous type of cercariae identified as* Diplostomum pseudospathaceum *was reported at a rate of 18.8% in the same species of* Lymnaea* [46].

The abundant presence of these non-*Fasciola *cercariae in the local lymnaeid snails may control* Fasciola *spp. transmission as governed by larval competition and colonization, but may be difficult to establish in this current study. On the other hand, gymnocephalus cercaria of* Fasciola *spp. was found also among nonlymnaeid snails in other studies. These included* Bithynia siamensis *of the family Bithyniidae [47] and array of other snail genera like* Biomphalaria*,* Bulinus*,* Ceratophallus*,* Gabbiella*,* Gyraulus*, and* Melanoides* [27]. None as of this date has reported presence of this type of cercariae among snails other than* Lymnaea *spp. in the Philippines.

The results of the current study are not consistent with those of other studies. Devkota et al. [48] showed only gymnocephalus cercaria, which is typical of* Fasciola *spp. in 2.9% of the* Lymnaea luteola *snails studied, while Luka and Mbaya [49] found distome cercaria of* Fasciola *spp. in* Lymnaea natalensis*. Imani-Baran et al. [28], on the other hand, showed xiphidiocercariae frequenting their sampled* Lymnaea gedrosiana *snails at 81.98%, followed by furcocercariae (32.26%), echinostome cercariae (5.19%), and a monostome cercaria which belongs to the family Notocotylidae (1.24%). Ramitha and Vasandakumar [1] likewise recovered furcocercous, echinostome cercariae, and xiphidiocercariae inhabiting* L. luteola. *Presence of these varieties of cercariae clearly is a manifestation of a wide array of snail-parasite interaction and symbiosis. This abundance of trematode species that exploit snails as their mandatory first intermediate host has also been documented and reviewed in Europe [50]. In fact, richness of malacofauna in the area also demonstrated parasite diversity as shown commonly by various larvae infecting a single snail host one at a time or rarely by the occurrence of multiple infections.

There are ten different possible cercariae that may be recovered from lymnaeid snails [27], but only three types were found inhabiting the local lymnaeids in Batangas. Most were nonzoonotic parasites. However, the presence of echinostome type and forked-tailed cercariae may be explored further as these two are known to be medically important.* Echinostoma *spp. are intestinal parasites of humans [51], and fork-tailed cercariae of avian origin can cause dermatitis. Echinostomes also are known to heavily infect domestic ducks, thus affecting greatly poultry business [52]. In the Philippines, two medically important echinostomes,* Echinostoma ilocanum *and* Echinostoma malayanum*, were recovered from infected humans that required freshwater gastropods belonging to the family Lymnaeidae as intermediate hosts [32].

Virgulate xiphidiocercaria is common among snails belonging to the genera* Lymnaea *and* Melanoides* [25], but was also reported in the genus* Thiara *[53]. They are known to be produced by intestinal parasites belonging to the family Lecithodendriidae which may infect animals like bats, birds, and amphibians. Poultry animals like ducks and chickens were observed in the perimeter of the sampling site and may provide a rationale for the existence of this type of cercariae. No studies have been made on their pathology in these animals. Therefore, its veterinary impact on poultry animals is yet to be explored.

Fork-tailed cercariae have never been reported in the Philippines to occur in snails other than* Oncomelania hupensis quadrasi*, the local snail hosts for* Schistosoma japonicum*. Longifurcate- pharyngeate distome cercariae (strigeid) were the second most common cercariae in the samples collected and were twice as long as the cercariae of* Schistosoma japonicum. *The size of the snail host was also twice longer than the* Oncomelania *snail. Several snail hosts can harbor any of the following different types of fork-tailed cercariae: brevifurcate-apharyngeate distome cercariae, brevifurcate-apharyngeate monostome cercariae, longifurcate-pharyngeate distome cercariae, and longifurcate-pharyngeate monostome cercariae. These snails include the following genera:* Biomphalaria*,* Bulinus*,* Ceratophallus*,* Cleopatra*,* Gabbiella*,* Gyraulus*,* Melanoides*,* Melanopsis*,* Segmentorbis *[27],* Indoplanorbis*, and* Bithynia* [47]. Among these, the brevifurcate-apharyngeate distome cercaria has been given medical attention because of its public health impact. None to date has explored the presence of non-*Schistosoma *sp. fork-tailed cercariae in these wide array of snail intermediate hosts. The possibility of recovering avian schistosomes causing dermatitis-like conditions from the sampling site is high because birds and other poultry animals abound in the area. This has yet to be confirmed.

Encysted forms were observed as a result of light exposures over time. This natural mechanism of encystment is a protective response on the parasite which was exposed to unconducive environment [41]. Cercarial release in the absence of suitable second intermediate host from a given environment leads to death of the larval stage. In this context, encysted forms are mostly observed among snails that have been exposed to light longer during cercarial emergence. However, identity of the encysted forms was difficult to establish but they may belong to any one of the three recovered types of cercariae: echinostome, xiphidiocercaria, or the strigeid.

### 4.3. Existence of Double Infection in a Single Lymnaeid Snail

Although single trematode parasitism is commonly demonstrated by majority of snail infection cases, very few have noted coinfection in other snail species like that of* Indoplanorbis exustus, *where xiphidiocercaria and longifurcate-pharyngeate distome cercaria existed in a single snail host [48]. Once a parasite inhabits a snail host, it initiates chemical changes that alter the host's attractiveness to other invading parasites [54]. From there, it establishes a territory of its own and creates its progenies within. Thus, any deviation from this is a very rare phenomenon and is likely due to simultaneous infection by two trematode species [54]. However, other factors like chronological and spatial variations in the number of eggs and miracidia limit the possibility of simultaneous multiple trematode infections [55, 56].

Here in the Philippines, the finding of coinfection in our lymnaeid snails with echinostome cercariae and fork-tailed cercariae (0.27%) and echinostome with xiphidiocercaria (0.13%) is first in the recorded history which can be either a coincidence or a real coexisting phenomenon. Density of each type of coexisting cercariae was not determined, so the degree of competition between them could not be duly determined. Moreover, the very low occurrence may be due to increased snail mortalities which are associated with increased pathogenicity of double infections as compared to single infection [57]. Consequently, the sample snails demonstrating simultaneous multiple infections may be underrepresented. According to Sewell [58], only certain combinations of trematode species could coexist together as double infections, and these double infections generally involved the following cercarial groups: furcocercariae, xiphidiocercariae, and monostome cercariae. These findings are consistent with our current results except for the existence of echinostome cercariae in the pair. It may seem incidental, but if a given snail is home for a wide spectrum of trematode species, there is likelihood that coinfection happens such as in the case of* B. siamensis* [47].

### 4.4. Snail Infection Rates in Different Months of Collection

Monthly variations in snail infection rates were observed over the five-month collection period. Lowest infection rate was seen in December 2016 (21.3%), and the highest was in February 2017 (57.3%) with echinostome type of cercariae frequenting the infected samples. No trend was observed over the 5-month period. The differences may be attributed to crop rotation and field practice of the nearby residents. Periodic sprays with chemicals against mollusc pests affect snail density and therefore may affect parasite transmission from one host to another. Monthly variations in infection rates may have a trending pattern if practices of residents are controlled and variations are all attributable to weather conditions. As such, more snails will be expected to be infected during rainy season as compared to those collected during summer [59–61].

### 4.5. Snail Morphometrics and Infection Rates

Changes in morphology are associated with snail's natural growth and development. It can also be an antipredatory adaptive mechanism among parasite-induced snail hosts [62]. Burch [63] showed that shell length provided a rough estimate of the age of the snail, and since this was positively correlated with both shell width (*r *= 0.797) and weight (*r *= 0.743), it was deduced that any of these three morphological features could be employed to determine the age of every snail. In fact, a highly significant correlation of these morphometric traits (shell length and shell width:* r *= 0.757; shell length and total live weight:* r *= 0.770;* p*<0.01) was established among* L. acuminate* [64].

Our current findings showed that 35.6% of sampled snails that were naturally infected were longer [OR = 1.809; 95% CI: 1.471–2.225;* p*<0.001] as compared to the uninfected group. Length coincides with the age of snails [65]. Although resistance to infection is observed among older snails [25], they may have been exposed to a number of miracidia as they mature and may have been infected for a longer period. Snail infection acquired at younger stages persists over time as snails develop [64]. Thus, increasing prevalence or intensity of infection is associated with increasing size vis-à-vis age of the snails [66–68]. Trematode-induced morphological changes can provide various consequences in the infected snail hosts: enhanced growth rate [69], stunted or decreased growth rate [70], and still or no effect in growth [71].

On the other hand, results of the study showed that gross weight of empty shells differed slightly between the infected and uninfected snails [average shell weight (infected) = 0.11 ± 0.01 > average shell weight (uninfected) = 0.09 ± 0.01]. However, logistic regression of the raw data showed that, statistically, the heavier the snail's shell, the lower the chance that it was infected. The state of calcification process, whether hypercalcification or hypocalcification, may provide a plausible and objective association to the state of infection, but this cannot be used to explain the results because the study did not measure calcium concentration of the empty shell. The degree of calcium utilization and subsequent deposition on the shell is a parasite type-dependent occurrence. In several reports, either shell hypercalcification [72, 73] or shell hypocalcification [74, 75] resulted after trematode-induced infection in selected snail hosts. In other words, the type of infecting parasite is an influential factor in calcification phenomenon in which this study did not determine anymore.

## 5. Conclusion

Lymnaeid snails that inhabit rice fields and man-made water ducts are potential carriers of zoonotic parasites that have both medical and veterinary public health significance. In this study, the total cercarial shedding rate of the snails as a measure of the infected snails was found to be 35.6%. Although single trematode infection was the common observation, coinfection with two different larval forms was found in less than 1% of the lymnaeid snails. The presence of any of the three different cercariae, i.e., echinostome, fork-tailed strigeid, and xiphidiocercariae, in the lymnaeid snails reflects the diversity of parasites and possibly of reservoir hosts, which may significantly pose potential health hazards to man and animals. Therefore, wide-scale surveillance of freshwater snails is necessary, including a catalogue on the variety of cercariae and their associated definitive hosts, to justify the necessity of control measures against snail-borne parasitic infections.

## Figures and Tables

**Table 1:** Types of cercariae and the frequency of recovery from lymnaeid snails (n=750).

**Type of Cercariae**	**Frequency**	%
*Single Infection*		
(i) Echinostome	198	26.4%
(ii) Virgulate Xiphidiocercaria	5	0.67%
(iii) longifurcate-pharyngeate distome cercariae (Strigea)	17	2.27%

*Mixed infection*		
(i) Echinostome and fork-tailed cercariae (Strigea)	2	0.27%
(ii) Echinostome and Virgulate xiphidiocercaria	1	0.13%

Encysted forms only	44	5.9%

**Table 2:** Number of infected snails in the different months of collection and after different light exposure periods as monitored by cercarial shedding.

	**Number of snails collected**	**Number of Infected snails**	**Odds Ratio (95**%** CI)**
**Total Number**	**750**	**267 (35.6**%**)**	

**∗** **Month of Collection**			
November 2016	150	45 (30.0%)^c^	-
December 2016	150	32 (21.3%)^c^	0.63 (0.38 – 1.07)
January 2017	150	65 (43.3%)^a^	1.78 (1.11 – 2.87)
February 2017	150	86 (57.3%)^b^	3.14 (1.95 – 5.05)
March 2017	150	39 (26.0%)^c^	0.82 (0.50 – 1.36)

**∗** **∗** **Number of Light Exposures**			
One	150	44 (29.3%)	-
Two	150	55 (36.7%)	1.40 (0.86 – 2.26)
Three	150	55 (36.7%)	1.40 (0.86 – 2.26)
Four	150	56 (37.3%)	1.44 (0.89 – 2.33)
Five	150	57 (38.0%)	1.48 (0.91 – 2.39)

*∗*Each exposure group was composed of a total of 150 snails from 5 months of collection (n=30 snails/exposure/month).

*∗∗* Number of light exposures represents the number of times a given batch of snails received a 6-hour light exposure with an average ~1000 lux light intensity. There were no significant differences among the numbers of infected snails in the different treatment groups (p>0.05)

Values with the same letter are not significantly different from each other (p>0.05). Values with different letters are significantly different from each other (p<0.001).

**Table 3:** Shell length, width, and weight of infected and not infected samples.

	**Infected**	**Not Infected**	**Odds Ratio (95**%** CI)**
Number of Samples	267 (35.6%)	483 (64.4%)	-
Length (mm)	13.68 ± 0.09	12.54 ±0.08	1.809 (1.471 – 2.225)
Width (mm)	7.59 ± 0.06	7.07 ± 0.05	0.937 (0.672 – 1.305)
Shell Weight (g)	0.11 ± 0.01	0.09 ± 0.01	0.003 (0.000 – 0.275)

Values expressed as counts (%) and mean ± SEM.

**Table 4:** Counts of snails with encysted forms based on number of light exposures.

Number of Light Exposures	Number and % Snails with Encysted Form (n=150)	Odds Ratio (95% CI)
One	3 (2.0%)^a^	-
Two	8 (5.3%)^a^	2.76 (0.72 – 10.62)
Three	21 (14.0%)^b^	7.98 (2.33 – 27.36)
Four	21 (14.0%)^b^	7.98 (2.33 – 27.36)
Five	26 (17.3%)^b^	10.27 (3.04 – 34.76)

Values expressed as counts (%).

*∗*Each exposure group was composed of a total of 150 snails from 5 months of collection (n=30 snails/exposure/month).

*∗* Number of light exposures represents the number of times a given batch of snails received a 6-hour light exposure with an average ~1000 lux light intensity.

Values with the same letter are not significantly different from each other (p>0.001). Values with different letters are significantly different from each other (p<0.001).

## Data Availability

The data used to support the findings of this study are included within the article.

## Supplementary Materials

Supplementary Materials Supplemental Figure 1: illuminated cabinet devised and used for cercarial emergence. Distance of light source to the top surface of the specimen container is 35 cm. Set-up includes apparatus for temperature and relative humidity determination and a lux meter for measuring light intensity.
