# Supplementary material for: Morphological Characterization of Emerging Cercariae among Lymnaeid Snails from Barangay Cawongan, Padre Garcia, Batangas, Philippines
Source: J Parasitol Res. 2018 Sep 10;2018:5241217. doi: 10.1155/2018/5241217 (PMC6151677; doi:10.1155/2018/5241217)
Supplement: Supplementary Materials — Supplemental Figure 1: illuminated cabinet devised and used for cercarial emergence. Distance of light source to the top surface of the specimen container is 35 cm. Set-up includes apparatus for temperature and relative humidity determination and a lux meter for measuring light intensity. [file 5241217.f1.pdf]

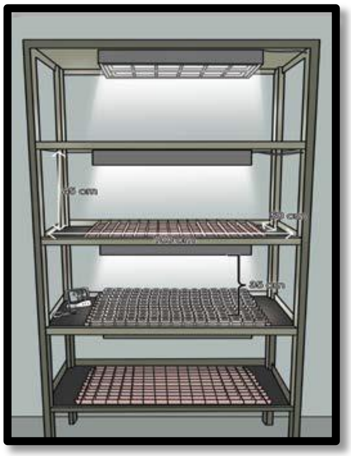

Supplemental Figure 1. Illuminated cabinet devised and used for cercarial emergence. Distance of light source to the top surface of the specimen container is 35 cm. Set-up includes apparatus for temperature and relative humidity determination and a lux meter for measuring light intensity.
